# Dr. Long and the Hall of Fame

**Published:** 1902-05

**Authors:** L. B. Grandy

**Affiliations:** Major and Surgeon, U. S. Vols.; Military Hospital, Lipa, Batanzas Province, Luzon, P. I.


### CORRESPONDENCE.

---

### DR. LONG AND THE HALL OF FAME.

Military Hospital,
Lipa, Batanzas Province, Luzon, P. I.,
February 26th, 1902.

*Editor Atlanta Journal-Record of Medicine:*

A letter received some days ago from a medical friend in Baltimore informs me that there is to be a reconsideration of the selection of Dr. Crawford Long as one of the Georgians to be honored with a statue in the "Hall of Fame" at Washington. He states that a committee of the Legislature has been appointed to review this whole matter, and apparently there is some fear that someone else may be selected.

I thought this question was closed long ago. When the portrait of Dr. Long, now in the Capitol at Atlanta, was presented to the State of Georgia by a wealthy philanthropist (whose name I cannot now recall) in commemoration of the discovery of anesthesia, Mr. Stephens, in an eloquent address advised that Georgia's two places in Statuary Hall, Washington, be reserved for statues of Oglethorpe, the first governor and Long the discoverer of anesthesia, and this suggestion was adopted and made official, I believe, by vote of the Legislature. This was about 1879, I think. (I am writing this from memory entirely and without means of verifying these impressions.) So up to that point the case appears to be closed.

Why is it now necessary to reopen this matter, and why does anybody wish to set aside Dr. Long for some other Georgian? Is his memory any less worthy of being honored now than it was twenty years ago? What has transpired in the interim to lessen the priceless value of his discovery to mankind or to diminish the honor so justly due him for poineer work in surgical anesthesia sixty years ago? Or does the Legislature of to-day contend that the Legislature of 1879 was not competent to make judicious selections?

Every day since 1877, when Dr. Long's priority in the use of ether for surgical purposes was brought prominently before the medical profession by Dr. Marion Sims, has only served to diffuse the knowledge of his work and name. The matter has been taken up where Sims left off by a number of medical writers as a labor of love, both at home and abroad. I have at home a large scrap-book filled with clippings and articles upon Dr. Long's priority in the discovery of anesthesia. These include communications to the medical press from persons under the very shadow of the ether monument in Boston. One distinguished English surgeon, Dr. Foy, of Dublin, has been an able advocate of Dr. Long in the English and German medical press.

What a spectacle to the outside world if this Legislative endorsement of Dr. Long's genius should now be repudiated! What a shame and humiliation upon the Georgia medical profession! Some of us have endeavored to keep Dr. Long's work before the medical world by arguing his cause whenever occasion presented, and our efforts have met with encouraging success; but no amount of argument and no process of reasoning in the future could break down the solitary fact that while one Legislature of Dr. Long's own state recognized his genius and wished to honor him for his noble and unselfish benefactions he was cruelly repudiated by another.

We hear a good deal in Georgia about Dr. Long's discovery of anesthesia, and any body there who knows anything about the history of this matter will bravely champion his cause when occasion offers, but what concerted action has ever been taken that has ever produced any results? Societies have resolved this thing and the other thing and committees have investigated and reported so and so to the unanimous satisfaction of these bodies, but after all what has been actually performed in Dr. Long's own State? The portrait of Dr. Long above mentioned was presented to the State *by a Northern man.* Three years ago I wrote a letter to the secretary of the State Medical Association saying that I understood some movement would be initiated at the approaching meeting to raise funds for a monument to Dr. Long; that if anything was done in this direction I was authorized by a friend of mine and of the Long family, who wished name witheld, to subscribe a liberal sum (I cannot recall now the exact amount, as this proposition is in writing at home; it was $100.00 or $200.00, I think), but I have never heard anything further of such a movement or been asked for the money.

In Boston there is a beautiful monument to Dr. Morton, and in Hartford another to Dr. Wells. Each claimed the honor of the discovery of anesthesia, and the claim is duly maintained on their tombs. Long antedated Wells by two years and Morton by four years, and had several times operated successfully under ether before either of these gentlemen had discovered anything. There used to be an old gentleman in Edgewood, near Atlanta, named Goodman, who as druggist furnished Dr. Long with his ether (he may be dead now, though I don't know).

I hope that the doctors who read this letter will exert their influence where it will be most useful, to the end that this reproach of apathy and indifference may no longer rest against us. Then may the State's original intentions be carried out and we may see the most important medical event of the nineteenth century duly commemorated in Washington, with a modest young practioner of Georgia as the real hero.

L. B. GRANDY, M.D.,
Major and Surgeon, U. S. Vols.

---

ACOINE is the name of a product destined to rival cocaine, morphine, chloral and other anesthetics. A drop upon a gnawing tooth diminishes pain. Its properties were recently reported to the French Academy of Medicine by Dr. Chauvel, based upon experiments. It is claimed that acoine is not toxic.—*Exchange.*

